# Genetic Isolation among the Northwestern, Southwestern and Central-Eastern Indian Ocean Populations of the Pronghorn Spiny Lobster *Panulirus penicillatus*

**DOI:** 10.3390/ijms15069242

**Published:** 2014-05-26

**Authors:** Muhamad Fadry Abdullah, Alimuddin, Mohamed Muththalib, Adnan Jameel Salama, Hideyuki Imai

**Affiliations:** 1 Graduate School of Engineering and Science, University of the Ryukyus, Nishihara, Okinawa 903-0213, Japan; 2 Faculty of Fisheries and Marine Science, Bogor Agricultural University, Bogor 16680, Indonesia; E-Mail: alimuddin_alsani@yahoo.com; 3 Ministry of Fisheries and Agriculture, Malé 20125, Maldives; E-Mail: mohamedmuththalib@gmail.com; 4 Faculty of Marine Science, King Abdulaziz University, Jeddah 21589, Saudi Arabia; E-Mail: ajsalama@kau.edu.sa; 5 Faculty of Science, University of the Ryukyus, Nishihara, Okinawa 903-0213, Japan; E-Mail: imai@sci.u-ryukyu.ac.jp

**Keywords:** pronghorn spiny lobster, *Panulirus penicillatus*, genetic diversity, population structure, Indian Ocean

## Abstract

The pronghorn spiny lobster *Panulirus penicillatus* is a highly valuable species which is widely distributed in Indo-West Pacific and Eastern Pacific regions. Mitochondrial DNA control region sequences (566–571 bp) were determined to investigate the population genetic structure of this species in the Indian Ocean. In total, 236 adult individuals of *Panulirus penicillatus* were collected from five locations in the Indian Ocean region. Almost all individuals had a unique haplotype. Intrapopulation haplotype (*h*) and nucleotide (*π*) diversities were high for each locality, ranging from *h* = 0.9986–1.0000 and *π* = 0.031593–0.043441. We observed distinct genetic isolation of population located at the northwestern and southwestern edge of the species range. Gene flow was found within localities in the central and eastern region of the Indian Ocean, probably resulting from an extended planktonic larval stage and prevailing ocean currents.

## 1. Introduction

The pronghorn spiny lobster *Panulirus penicillatus* is widely distributed in the Indo-West Pacific and Eastern Pacific regions. It is found in tropical and adjacent regions from South-Eastern Africa, the Red Sea, Southern India, the Southeast Asian Archipelago, Japan, Northern Australia, and the Southern and Western Pacific Islands, to Hawaii, the Galápagos Islands, and other islands of the Eastern Pacific [1,2,3]. *P. penicillatus* supports considerable fisheries in Indo-Pacific regions. The species is fished wherever it occurs. Much attention has been paid to biological investigation of young and adult stages. Recognition of stocks is complicated by the life cycle of pronghorn spiny lobster, primarily because of the potential for long-range dispersal during its planktonic larvae. The early life history of spiny lobsters consists of a drifting larval period adapted for a relatively long-term stay in the open ocean, extending from several months to more than a year, with many possibilities for dispersal through ocean currents [4,5].

Several molecular techniques, including allozymes, restriction fragment length polymorphism (RFLP), mitochondrial DNA sequencing, and microsatellites, have been used in studying genetic variability in marine organisms at the population level [6,7]. Mitochondrial DNA sequencing, particularly of the most rapidly evolving and highly variable control region, has been a useful tool for population genetic studies of many terrestrial and aquatic organisms [8]. The highly polymorphic mitochondrial control region sequence has been used previously for population genetics studies in spiny lobster species [9,10,11,12].

There recently been studies of *P. penicillatus*, East-West Pacific differentiation using 16s and COI (cytochrome oxidase I) marker was described by Chow *et al.* [13], and this study was corroborated with the study of genetic population structure of *P. penicillatus* in the Pacific region using highly polymorphic marker, mitochondrial control region by Abdullah *et al.* [3]. In this study, we focused on the Indian Ocean region of *P. penicillatus*.

**Figure 1:**
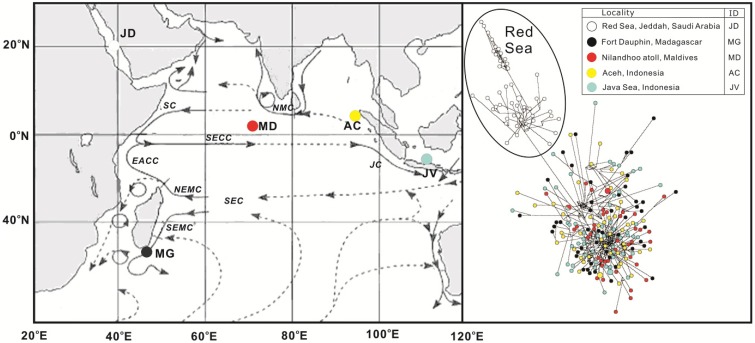
Map of the Indian Ocean (**left**): showing SEC—South Equatorial Current, SECC—South Equatorial Countercurrent, SEMC (NEMC)—Southeast (Northeast) Madagascar Current, NMC (SMC)—Northeast (Southwest) Monsoon Current, JC—South Java Current, ocean current was drawn referring to Schott and McCreary [14]. *Panulirus penicillatus* haplotype network (**right**); Sizes of the circles are proportional to the frequency of each haplotype; lengths of the lines are relative to the number of mutations between haplotypes.

There have been no previous population genetic studies of *P. penicillatus* in the Indian Ocean region. The aim of the present study is to investigate population genetic structuring in a widely distributed *P. penicillatus* in the Indian Ocean region. We address this question using mitochondrial control region sequences data from Red Sea, Jeddah, Saudi Arabia; Fort Dauphin, Madagascar; Nilandhoo atoll, Maldives; Aceh and Java Sea, Indonesia (Figure 1). Given the economic importance of *P. penicillatus*, this genetic information may be valuable for long-term fisheries’ management decisions.

## 2. Results and Discussion

### 2.1. Genetic Diversity Analysis

A total of 566–571 bp sequence data were obtained from 236 specimens of *P. penicillatus*. Of the 577 aligned base pairs, 246 variable sites (including indels) were found: 172 were parsimony informative and 74 were singletons. In total, 231 haplotypes were identified, and nearly all sequenced individuals had a unique haplotype (Table 1). The nucleotide sequences of the haplotypes were deposited in the DNA Data Bank of Japan (DDBJ) (accession numbers AB922639–AB922816, AB689451–AB689503).

As expected, this fragment is AT-rich in nucleotide composition with ratios of 37.8% adenine, 31.8% thymine, 19.4% cytosine, and 11% guanine, consistent with previous reports of an AT-rich control region of the mitochondrial genome in many invertebrates, including crustaceans [7,15].

The haplotype network showed a geographic structure with two main groups (Figure 1): (1) the northeastern edge of the population; Red Sea cluster and (2) other localities from the southwestern to eastern Indian Ocean (Madagascar, Maldives, Aceh and Java) that are closely together.

**Table 1:** Sample abbreviation (ID), sample locality, sample size (*N*), year of collection, numbers of haplotypes, haplotype diversity (*h*), nucleotide diversity (*π*), Tajima’s *D* and Fu’s *F* Test for *Panulirus penicillatus* mitochondrial DNA control region.

ID	Locality	*N*	Year	No. of Haplotypes (Unique)	*h* ± SD	*π* ± SD	Tajima’s *D* (*p-*Value)	Fu’s *F* (*p-*Value)
JD	Red Sea, Jeddah, Saudi Arabia	46	2011	45(45)	0.9990 ± 0.0048	0.031593 ± 0.015851	−0.79131 (0.215)	−24.20576 (0.000)
MG	Fort Dauphin, Madagascar	49	2014	49(49)	1.0000 ± 0.0041	0.043441 ± 0.021533	−0.97966 (0.168)	−24.14011 (0.000)
MD	Nilandhoo atoll, Maldives	38	2013	37(36)	0.9986 ± 0.0065	0.034957 ± 0.017561	−1.05781 (0.127)	−19.70407 (0.000)
AC	Aceh, Indonesia	48	2011	47(47)	0.9991 ± 0.0045	0.038100 ± 0.018974	−1.17981 (0.098)	−24.14392 (0.000)
JV	Java Sea, Indonesia	55	2008	53(52)	0.9993 ± 0.0036	0.038855 ± 0.019284	−1.01996 (0.153)	−24.12306 (0.000)
Total		236	-	-	-	-	-	-

Genetic diversity, as shown by haplotype diversity, was found to be very high in *P. penicillatus*. The values generated in this study are comparable with those derived from the mtDNA control region sequences for the crustaceans *Farfantepenaeus aztecus*, *Litopenaeus setiferus* and *Sergia lucens* [16,17]. Haplotype diversity (*h*) within the geographical populations was high, ranging from 0.9986 in Nilandhoo atoll, Maldives to 1.0000 in Fort Dauphin, Madagascar. Nucleotide diversity (*π*) was generally high, ranging from 0.031593 in Red Sea, Jeddah, Saudi Arabia to 0.043441 in Fort Dauphin, Madagascar (Table 1). There were few shared haplotypes among locations belonging to the central-eastern Indian Ocean.

In the present study, the mtDNA control region exhibited high genetic variability (*h* = 0.9986−1.0000 and *π* = 0.031593−0.043441), which are relatively high values compared with the control region diversities of other *Panulirus* spp. (*P. inflatus*: *h* = 0.957–1.0000 and π = 0.019–0.028 [10]; *P. argus*: *h* = 0.667–1.0000 and π = 0.003–0.066 [7], *P. homarus*: *h* = 0.985–1.0000 and π = 0.031–0.041 [12]) and similar to Pacific region populations of *P. penicillatus* (*h* = 0.9996–1.0000 and *π* = 0.0255–0.0448) [3]. The control region is a non-coding region that includes signals necessary for replication of a molecule and is the most rapidly evolving region of mtDNA [18].

The high genetic variability of the control region in *P. penicillatus* may be maintained by its large population size. In fact, because nearly all individuals represented a unique haplotype, an investigation based on sequencing the control region would require a very large sample size to detect common haplotypes and associate them with particular geographic areas [10]. The evolutionary pattern of interhaplotypic divergence will almost always produce a biogeographic signature, and an analysis that recognizes genetic distance among alleles will provide additional resolution, especially for hypervariable markers [19].

### 2.2. Population Structure Analysis

Genetic analysis of mitochondrial control region revealed distinct population at the northwestern and southwestern margin of geographic range of *P. penicillatus* with other localities. This suggests that the geographic isolation may be due to contemporary isolation/gene flow or prior isolation with recent gene flow, although the overall level of genetic exchange must be below that required to homogenize the populations.

Congruent with the pattern observed in the network haplotypes (Figure 1), we found strong regional structuring between Red Sea and other Indian Ocean populations of *P. penicillatus*, which explained high genetic variations (51%) between the regions. High genetic variation (85%) was also observed in previous study between eastern Pacific and west-central Pacific population of *P. penicillatus* [3]. To analyze the sub-structure in the other area we attempted three types of regional grouping: (1) Madagascar and Maldives/Aceh/Java; (2) Madagascar/Maldives and Aceh/Java; and (3) Madagascar/Aceh and Maldives/Java. Regarding these groupings, genetic variation was relatively greater between Madagascar and Maldives/Aceh/Java (2.41%) than the variations between other combinations (%var −0.49 and −0.75) (Table 2).

**Table 2:** AMOVA results showing degree of freedom (*df*), variance components (Var.), percent variation (% Var.) and *Φ*-statistics for *Panulirus penicillatus* (1000 permutations).

Source of Variation	*df*	Var.	% Var.	*Φ*-Statistics	*p*-Value
*All sites (Red Sea/Madagascar-Maldives-Aceh-Java)*					
Among groups	1	11.50819	51.31	*Φ_CT_* = 0.51313	*p* = 0.19746
Among populations	3	0.18886	0.84	*Φ_SC_* = 0.01730	*p* = 0.00098
Within populations	231	10.73030	47.84	*Φ_ST_* = 0.51313	*p* = 0.00000
*Substructure grouping test: (Madagascar-Maldives/Aceh/Java)*					
Among groups	1	0.27591	2.41	*Φ_CT_* = 0.02408	*p* = 0.24047
Among populations	2	0.03850	0.34	*Φ_SC_* = 0.00344	*p* = 0.14467
Within populations	186	11.14413	97.26	*Φ_ST_* = 0.02744	*p* = 0.00098
*(Madagascar/Maldives-Aceh/Java)*					
Among groups	1	−0.05540	−0.49	*Φ_CT_* = −0.00490	*p* = 0.67742
Among populations	2	0.21697	1.92	*Φ_SC_* = 0.01910	*p* = 0.00293
Within populations	186	11.14413	98.57	*Φ_ST_* = 0.01429	*p* = 0.00098
*(Madagascar/Aceh-Maldives/Java)*					
Among groups	1	−0.08441	−0.75	*Φ_CT_* = −0.00747	*p* = 1.00000
Among populations	2	0.23666	2.09	*Φ_SC_* = 0.02079	*p* = 0.00098
Within populations	186	11.14413	98.65	*Φ_ST_* = 0.01348	*p* = 0.00098

Central-eastern region (Maldives/Aceh/Java) samples were pooled, suggested by population tree, haplotype network and ocean division. In addition, our preliminary analyses of pairwise *Φ_ST_* comparisons of each data set found no heterogeneity as observed within samples from the central-eastern region (Maldives/Aceh/Java) (data not shown), although it was a limited *Φ_CT_* (Table 2, grouping Madagascar-Maldives/Aceh/Java). This supports the notion of a genetically homogeneous central-eastern region population that includes Maldives, Aceh and Java Sea, justified by combining the samples within the locality.

Populations of *P. penicillatus* in the Indian Ocean region do not represent a genetically homogenous assemblage. Pairwise *Φ_ST_* comparisons detected genetic structure between the central-eastern region (Maldives, Aceh, and Java) and other populations (Madagascar and Red Sea) (Table 3). A relatively small structure was found between the southwestern population (Madagascar) and central-eastern populations (Maldives/Aceh/Java) of *P. penicillatus* (*Φ_ST_* value = 0.02629, *p* < 0.005; see Table 3). This pattern is in agreement with was previously observed from this region in reef fish *Myripritis berndti* [20] and amphidromous prawn *Macrobrachium lar* [21]. The prevailing ocean currents in the southwest Indian Ocean splits the coast of Madagascar into the southeast Madagascar current and the northeast Madagascar current, which may have limited genetic exchange with other regions [21,22].

**Table 3:** Pairwise *Φ_ST_* values (above the diagonal) and pairwise *Φ_ST_ p*-values (below the diagonal) of mitochondrial DNA control region among populations of *Panulirus penicillatus*.

Population	Northwestern Region (Red Sea)	Southwestern Region (Madagascar)	Central-Eastern Region (Maldives-Aceh-Java)
Northwestern Region (Red Sea)	-	0.51058	0.53463
Southwestern Region (Madagascar)	0.00000 *	-	0.02629
Central-Eastern Region (Maldives-Aceh-Java)	0.00000 *	0.00098 *	-

* Significant *p*-value after Bonferroni correction (α = 0.05).

**Figure 2:**
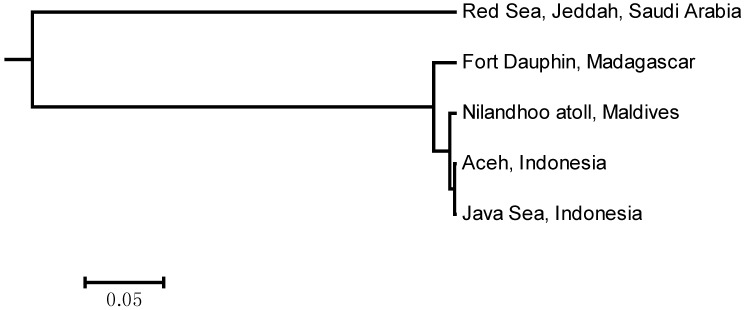
UPGMA tree based on *Φ_ST_* values, illustrating the most probable geographical structure in the analysis of molecular variance (AMOVA).

The greatest significance is found in northwestern region (Red Sea) with supported by significant and high pairwise *Φ_ST_* values (Table 3), UPGMA tree based on *Φ_ST_* values showed a prominent genetic break between Red Sea and other populations (Figure 2) this result also corroborated with the distinction of the Red Sea cluster in the haplotype network (Figure 1), other studies on invertebrates, mud crab *Scylla serrata* [23,24], have shown a genetic isolation of Red Sea populations from the Indian Ocean. Oceanographic currents are likely to create barriers and impact routes and directions of larval dispersal [25,26,27]. More importantly, the lack of shared mtDNA haplotypes and large *Φ_ST_* values suggest that there has been prolonged historical isolation between the Red Sea and others populations. In addition, high degree of endemism and biodiversity reef fishes were reported previously from the Red Sea, despite a geological history characterized by intermittent isolation and multiple salinity crises, mtDNA indicate that some widespread reef fish species originate in the Red Sea with subsequent contributing species to adjacent regions [28].

Further analysis on recruitment of *P. penicillatus* phyllosoma larvae may be necessary since genetic distinction in Red Sea populations may indicate the potential self-recruitment of phyllosoma larvae in this region. Although phyllosoma larvae are weak swimmers, they have the ability to maintain their vertical position in the water column [29]. Larval dispersal for spiny lobsters has been previously modeled. Griffin *et al.* [30], discovered that *P. cygnus* was a self-recruiting species, restricted to Western Australia. A more recent simulation study of *P. argus* found the average dispersal of *P. argus* in the Caribbean Sea to be possibly only 200–400 km, suggesting that larval behavior such as vertical migration, together with a retentive oceanographic environment, may increase the potential for self-recruitment [31].

No population structure was found in the localites of *P. penicillatus* inhabiting the central-eastern region of Indian Ocean: Nilandhoo atoll, Maldives; Aceh and Java Sea, Indonesia. The early life history of spiny lobsters consists of a drifting larval period adapted for a relatively long-term stay in the open ocean, extending from several months to more than a year, with many possibilities for dispersal through ocean currents [4,5]. Long larval life (8.3–9.4 months) of the *P. penicillatus* phyllosoma [32], coupled with the considerable impact of oceanic current systems or gyres (e.g., Equatorial currents and South Java current) (Figure 1) may explain the observed patterns. Furthermore, transportation of larvae likely occurred indirectly via stepping stones by the currents, and a small amount of larval dispersal over years might result in genetic homogeneity. Indeed, pelagic larval duration was considerably correlated with dispersal distance, but there were many exceptions; larval behavior can play a crucial role in determining dispersal distance [33], while type of eggs and life-history parameters are also important predictors of connectivity in fishes [27,34].

The neutrality of mutations in the mtDNA control region was rejected on the basis of Tajima’s *D* and Fu’ *F* tests. The significant negative values obtained in these *P. penicillatus* are often associated which experienced a population expansion during their geographic history [35]. This pattern has also been observed in other spiny lobster [12,36,37].

Although mtDNA has been proven to be a very useful marker, its use is not without complications. Due to the exclusively maternal inheritance mtDNA represents a marker strictly for historical process in females; they can potentially introgress between species [38,39], and symbiont-driven changes in mtDNA variation over space specifically in arthropods can occur [40]. Therefore, further studies incorporating supporting evidence from nuclear markers is required to obtain a more robust indication.

## 3. Experimental Section

### 3.1. Lobster Samples

Adults of *Panulirus penicillatus* were collected from five localities in the Indian Ocean region including Red Sea, Jeddah, Saudi Arabia; Fort Dauphin, Madagascar; Nilandhoo atoll, Maldives; Aceh and Java Sea, Indonesia (Figure 1; Table 1). Lobsters were purchased from local commercial fishers and fully complied with local fisheries management and marine protected area controls. Tissues samples from walking legs (pereiopods) or abdomen were dissected on site, immediately fixed in 70%–99% ethanol, and transferred to the laboratory.

### 3.2. DNA Analysis

About 50 mg of finely minced tissue sample was added to a 1.5 mL plastic test tube containing 0.5 mL TNES-8 M urea buffer [41]. After adding 10 μL proteinase K, it was incubated at 38 °C for 3 h and then genomic DNA was extracted using phenol-chloroform procedure and precipitated with absolute ethanol according method modified in Imai *et al.* [42]. The mtDNA control region was amplified using the polymerase chain reaction (PCR). PCR primers previously designed for mitochondrial control region (Panulirus12s: 5'-TATAGCAAGAATCAAACTATAG; and Penicillatus-R: 5'-CATAGG(T/C)GTG(T/C)GAGGGAACAAAGTC) were used for amplification of the control region [3]. PCR amplifications were performed in 50 μL reaction mixtures containing 1 μL template DNA, 12.5 pmol of each primer and 25 μL EmeraldAmp PCR Master Mix (2× Premix) (Takara Bio Inc., Shiga, Japan); the final volume of the reaction mixture was adjusted to 50 μL with sterile water. Reactions were performed in a thermal cycler (Perkin Elmer GeneAmp PCR System 9700) under conditions of an initial denaturation step at 94 °C for 2 min, followed by 30 cycles of 30 s at 95 °C, 30 s at 50 °C, and 1 min at 72 °C, with a final 7 min extension at 72 °C. PCR products were purified using a PCR Product Pre-sequencing kit (Exosap; USB Co., Cleveland, OH, USA). The cleaned products were sequenced on an ABI 3730xl Genetic Analyzer (Applied Biosystems, Foster City, CA, USA) using a Big Dye Terminator Cycle Sequencing kit (ver. 3.1; Applied Biosystems).

### 3.3. Genetic Data and Analyses

Sequence data were aligned using ClustalX [43], with default alignment parameters and were checked manually for misalignments. The nucleotide compositions and numbers of variable sites were assessed with MEGA6 [44]. Haplotype and nucleotide diversity for each location were estimated using Arlequin (ver. 3.5) software [45]. AMOVA were performed to test the geographic divisions among population experimented with various groupings. This approach is a hierarchical approach that computes the proportion of variations among groups (*Φ_CT_*), the proportion of variation among population within groups (*Φ_Sc_*), and the proportion of variation within populations (*Φ_ST_*). We ran preliminary analysis of pairwise *Φ_ST_* comparisons on each data sheet. The statistical significance of *Φ_ST_* value was tested by 1000 permutations in Arlequin (ver. 3.5) software [45]. The Bonferroni test [46] was used to correct for multiple tests of the hypothesis that pairwise *Φ_ST_* statistics did not differ from zero. An UPGMA tree based on Φ*_ST_* values was constructed using Neighbor in Phylip (ver. 3.6) [47,48]. The median-joining network [49], for the haplotypes was estimated using Network (ver. 4.611) [50]. Past demographic patterns were inferred by Tajima’s *D* [51], and Fu’s *F* [52], estimated using Arlequin (ver. 3.5). Both tests are commonly used to test neutrality; however, they can also be used to examine population growth because population expansion may result in rejection of the null hypothesis of neutrality (significant negative value).

## 4. Conclusions

The present study detected genetic structure in *P. penicillatus* populations located at the northwestern and southwestern edge of the Indian Ocean region with other populations. Relatively small structure was found between southwestern population and central-eastern, while vast majority structures were observed between the northwestern edge population and the other populations. Despite a lengthy larval period, oceanographic currents are likely to create barriers and impact routes and directions of larval dispersal in the northwestern edge population that may have limited genetic exchange with other regions. These results have implications for fisheries management in the region; this should essentially provide information for ascertaining stock boundaries to better evaluate the long-term needs of spiny lobster management for this valuable species.

At the scale of the central-eastern Indian Ocean, results suggested that *P. penicillatus* was belonging to a single unique panmictic population or at least several breeding grounds with significant exchange of genetic material. Long larval life (8.3–9.4 months) of the *P. penicillatus* coupled with the considerable impact of oceanic current systems or gyres (e.g., Equatorial currents and South Java current) may explain the observed patterns. Furthermore, transportation of larvae likely occurred indirectly via stepping stones by the currents, and a small amount of larval dispersal over some years might result in genetic homogeneity.
